# Harmonic memory signals in the human cerebral cortex induced by semantic relatedness of words

**DOI:** 10.1038/s41539-024-00221-1

**Published:** 2024-02-14

**Authors:** Yasuki Noguchi

**Affiliations:** Department of Psychology, Graduate School of Humanities, Kobe University, 1-1 Rokkodai-cho, Nada, Kobe 657-8501 Japan

**Keywords:** Human behaviour, Working memory, Language

## Abstract

When we memorize multiple words simultaneously, semantic relatedness among those words assists memory. For example, the information about “apple”, “banana,” and “orange” will be connected via a common concept of “fruits” and become easy to retain and recall. Neural mechanisms underlying this semantic integration in verbal working memory remain unclear. Here I used electroencephalography (EEG) and investigated neural signals when healthy human participants memorized five nouns semantically related (Sem trial) or not (NonSem trial). The regularity of oscillatory signals (8–30 Hz) during the retention period was found to be lower in NonSem than Sem trials, indicating that memorizing words unrelated to each other induced a non-harmonic (irregular) waveform in the temporal cortex. These results suggest that (i) semantic features of a word are retained as a set of neural oscillations at specific frequencies and (ii) memorizing words sharing a common semantic feature produces harmonic brain responses through a resonance or integration (sharing) of the oscillatory signals.

## Introduction

Verbal working memory (vWM) plays a critical role in various human behaviors such as reading, conversation, and inference, although its neural underpinnings remain controversial^1–3^. A hallmark of vWM is that its load can greatly change depending on a relationship among memory items^4^. For example, when multiple words in a memory list are semantically associated (“apple”, “banana”, and “orange”, etc.), the encoding and retention of those words is facilitated because they are integrated into a coherent concept (fruits) in the brain. In a typical experiment, this integration is evidenced by the high accuracy of a recognition task in which participants judge whether a probe word (e.g., “dog”, normally presented at the end of a trial) matches any of the memory words or not. In contrast to this adaptive aspect^5^, integrating memory items also has a negative effect, sometimes inducing an erroneous response in the recognition task^6–8^. If the probe is a lure word that is semantically related to memory items but has never appeared in the list (e.g., “pear”), typical participants falsely remember having seen the lure (called the “false memory” or “semantic interference”).

**Fig. 1:**
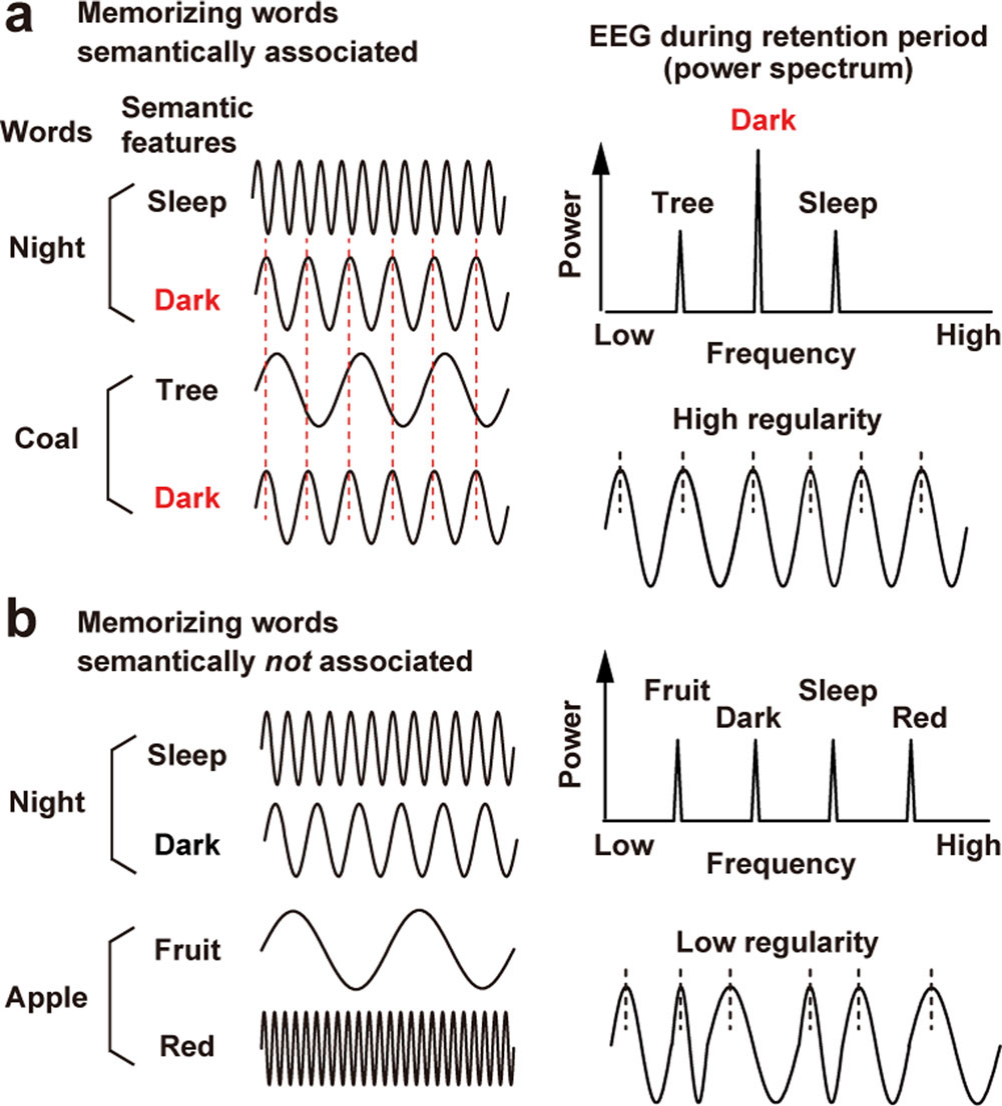
Scheme. For simplicity, here I assume that each semantic feature (e.g., sleep) of a word (e.g., night) is recalled as neural oscillation at a specific frequency. **a** Memorizing two words (e.g., night and coal) with a common feature (dark) induces a resonance or sharing of the oscillatory signal across the words, generating a regular EEG waveform characterized by a smaller number of frequencies in a power spectrum (right panels). **b** Memorizing words without a common semantic feature induces no resonance or sharing of the oscillatory signal, producing an irregular EEG waveform composed of a larger number of frequencies (like white noise).

Many studies have investigated brain activity underlying the semantic integration and interference in memory^9–12^. Approaches of functional magnetic resonance imaging (fMRI) and transcranial stimulation revealed critical brain regions such as the anterior temporal cortex^13,14^, prefrontal cortex^6^, and cerebellum^15^. However, it remained unclear how the semantic information is bound together as neural (electrical) signals in the human brain. Based on a close relationship between WM and oscillatory brain activities^16–21^, here I use electroencephalography (EEG) and test a hypothesis that a semantic integration in WM is represented as a harmony of neural rhythms (Fig. 1). In this model, semantic features of each memory word are maintained as a set of neural oscillations at specific frequencies. Retention of words sharing a common feature induces a resonance or integration of those oscillatory signals, generating a dominant frequency (or frequencies) in a power spectrum (Fig. 1a). This would produce a regular (more harmonic) neural waveform with a limited set of frequencies, forming a strong memory representation resistant to degradation by neural noises (irregular waveforms).

**Fig. 2:**
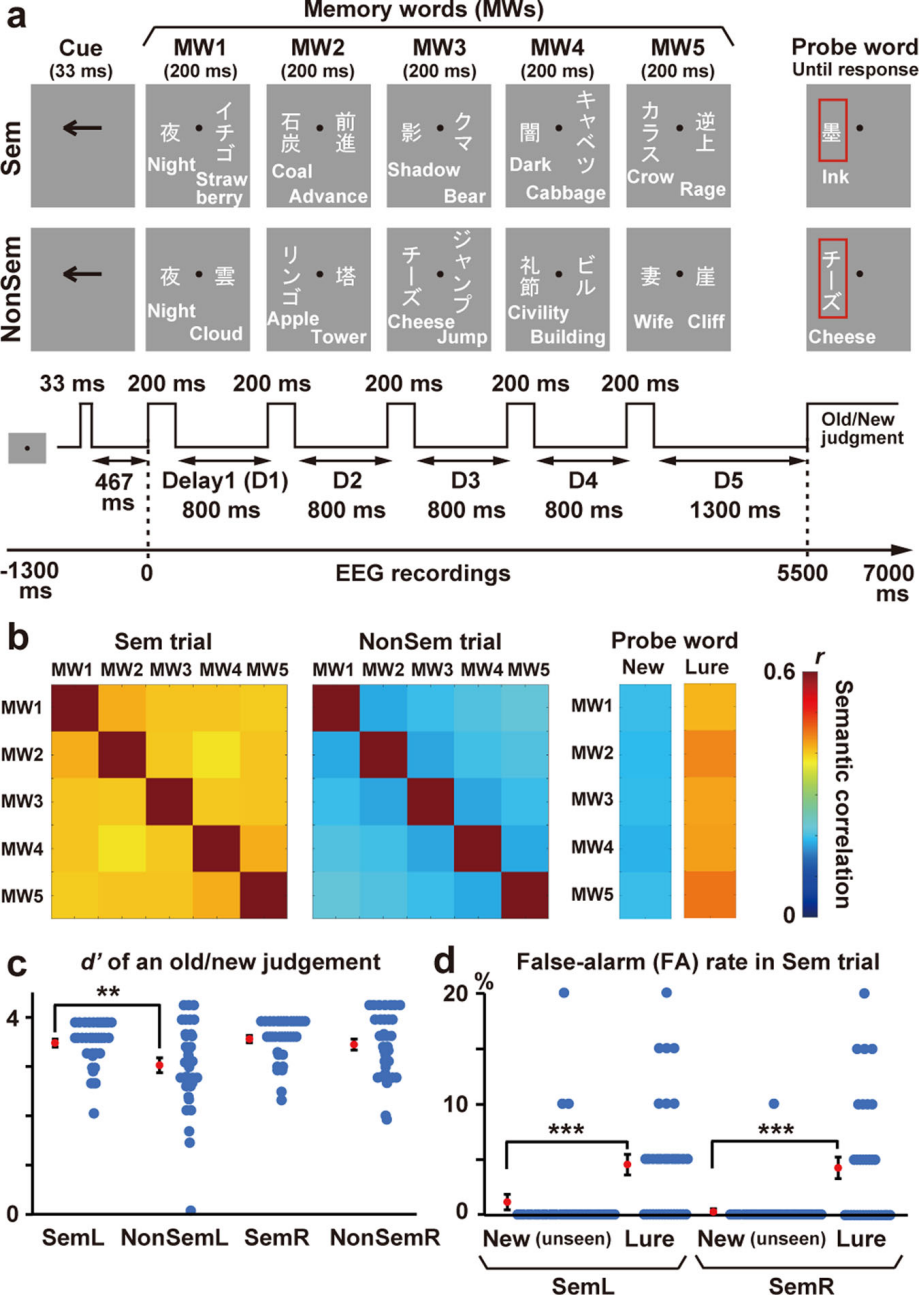
Experiment 1. **a** Each trial consisted of five screens (1 s/screen, containing two words for each), followed by a probe word marked with a red rectangle. Participants memorized 5 words in a visual field indicated by an arrow cue (memory words or MWs). They pressed one button if the probe matched any of the five MWs (“old” response) and pressed another if not (“new” response). Neural activity during a retention period after the 5th word (D5) was compared between when the MWs were semantically associated (Sem trial, upper panels) or not (NonSem trial, lower panels). **b** Semantic relatedness among five MWs in Sem (left) and NonSem (middle) trials. The relatedness between two words was computed as a correlation between semantic vectors (1 × 300) of those words. Semantic correlations between MWs and two types of probe words in Sem trials (unrelated new words and lure words, see Methods) are also shown in the right panels. **c** The d-prime (*d’*) computed from hit and false-alarm (FA) rates in the old/new judgment task. Participants showed better performance (higher *d’*) in Sem than NonSem trials only when they memorized MWs in the left visual field (Retain-Left conditions, SemL and NonSemL). **d** False memory. Higher FA rates to lure probes than new (unseen) probes were observed both in SemL and SemR trials. Blue and red dots show individual data and an across-participant average, respectively. Error bars denote standard errors. ** *p* < 0.01, *** *p* < 0.001.

As shown in Fig. 2a, participants in the present study attended to either the left or right visual field and memorized five words sequentially presented. Neural activity during a retention period after the 5th word (delay 5 or D5) was compared between when the five words were semantically associated (Sem trial) or not (NonSem trial). If an across-word integration takes place as a neural harmony in the brain, this would be observed as a higher regularity of EEG waveforms in Sem than NonSem trials. Specifically, EEG signals in Sem trials would be characterized by a smaller number of local peaks on a power spectrum density (PSD, upper right of Fig. 1a). In contrast, the PSD in NonSem trials would comprise a larger number of different frequencies (like a white noise), leading to an irregular EEG waveform where fast and slow oscillations are mixed together (Fig. 1b).

## Results

### Behavioral data (Experiment 1)

The basic structures of trials are displayed in Fig. 2a. Each trial started with a cue stimulus (an arrow) to direct the attention of participants. Participants (34 native speakers of Japanese) viewed two Japanese words (nouns), one in the left and another in the right visual fields, presented simultaneously for 200 ms (memory-word screen). A total of five screens (ten words) were sequentially presented with an inter-screen interval (delay) of 800 ms. After the last (5th) delay of 1300 ms, the trial ended with a probe word marked by a red rectangle. Participants memorized five words in the cued hemifield (memory words or MWs) and performed an old/new judgment on the probe word, pressing one button if the probe matched any of the five MWs (“old” response) but another if not (“new” response). They were also asked to ignore any words in an uncued hemifield.

An effect of semantic integration was measured by a comparison of Sem and NonSem trials (Fig. 2a, b). In Sem trials, five MWs in the cued hemifield were semantically related (e.g., “night”, “coal”, “shadow”, “dark”, and “crow”), while they were not in NonSem condition (e.g., “night”, “apple”, “cheese”, “civility”, and “wife”). A combination of cued hemifield (left/right) and semantic relatedness (Sem/NonSem) produced four types of trials randomly intermixed in an experimental session. Trials with a leftward cue and semantically related MWs were called SemL, while those with a rightward cue and unrelated MWs were called NonSemR.

In SemL and SemR, one of five MWs was shown as a probe in 20 out of 60 trials (old-probe trial, e.g., “night” in the above case). A probe word not included in MWs was shown in another 20 trials (new-probe trial, e.g., “foot”). In the other 20 trials, I showed a probe that was semantically related to the MWs but had never appeared (lure probe, e.g., “ink”). In NonSemL and NonSemR, 30 trials had an old probe, and the other 30 trials had a new probe.

Behavioral data were analyzed using the signal detection theory. I computed the d-prime (*d’*) based on a hit rate in which participants answered “old” to an old probe and a false-alarm (FA) rate in which they answered “old” to a new probe. If semantic relatedness across MWs facilitated the old/new judgment, this would be observed as higher *d’* in Sem than NonSem conditions. Semantic integration, however, is also known to exert a negative influence when the probe is related to memory words (false memory). I examined this point by comparing the FA rates between the lures and new (unseen) probes of the Sem condition. False memory would be indexed by a higher FA rate to lures than that to new probes.

Figure 2c shows the *d’* of the old/new judgment task. I observed the *d’* significantly higher in SemL (3.48 ± 0.08, mean ± SE across participants) than NonSemL (3.03 ± 0.15) trials (*t*(33) = 3.34, *p* = 0.002, Cohen’s *d* = 0.63), showing that semantic relatedness among MWs facilitated a retention of those words when they were presented in left visual field (right hemisphere). No significant difference, in contrast, was seen (*t*(33) = 1.05, *p* = 0.30, *d* = 0.20) when participants memorized words in the right visual field (SemR: *d’* = 3.56 ± 0.07, NonSemR: *d’* = 3.45 ± 0.11).

Figure 2d displays FA rates for new (unseen) probes and lure probes. In SemL, the FA rates to lures (4.56 ± 0.93%) were significantly higher (*t*(33) = 4.04, *p* = 0.0003, *d* = 0.70) than those to new words (1.18 ± 0.70%), showing a false memory arising from a semantic integration. Similar results were observed in SemR (lures: 4.26 ± 0.97%, new words: 0.29 ± 0.29%, *t*(33) = 4.34, *p* = 0.0001, *d* = 0.95)

Taken together, those data indicated that participants integrated semantic information of MWs irrespective of whether they were presented in the left or right visual field. A left-hemispheric dominance of vWM^22,23^, however, might obscure a difference in *d’* between SemR and NonSemR (ceiling effect).

### EEG data

**Fig. 3:**
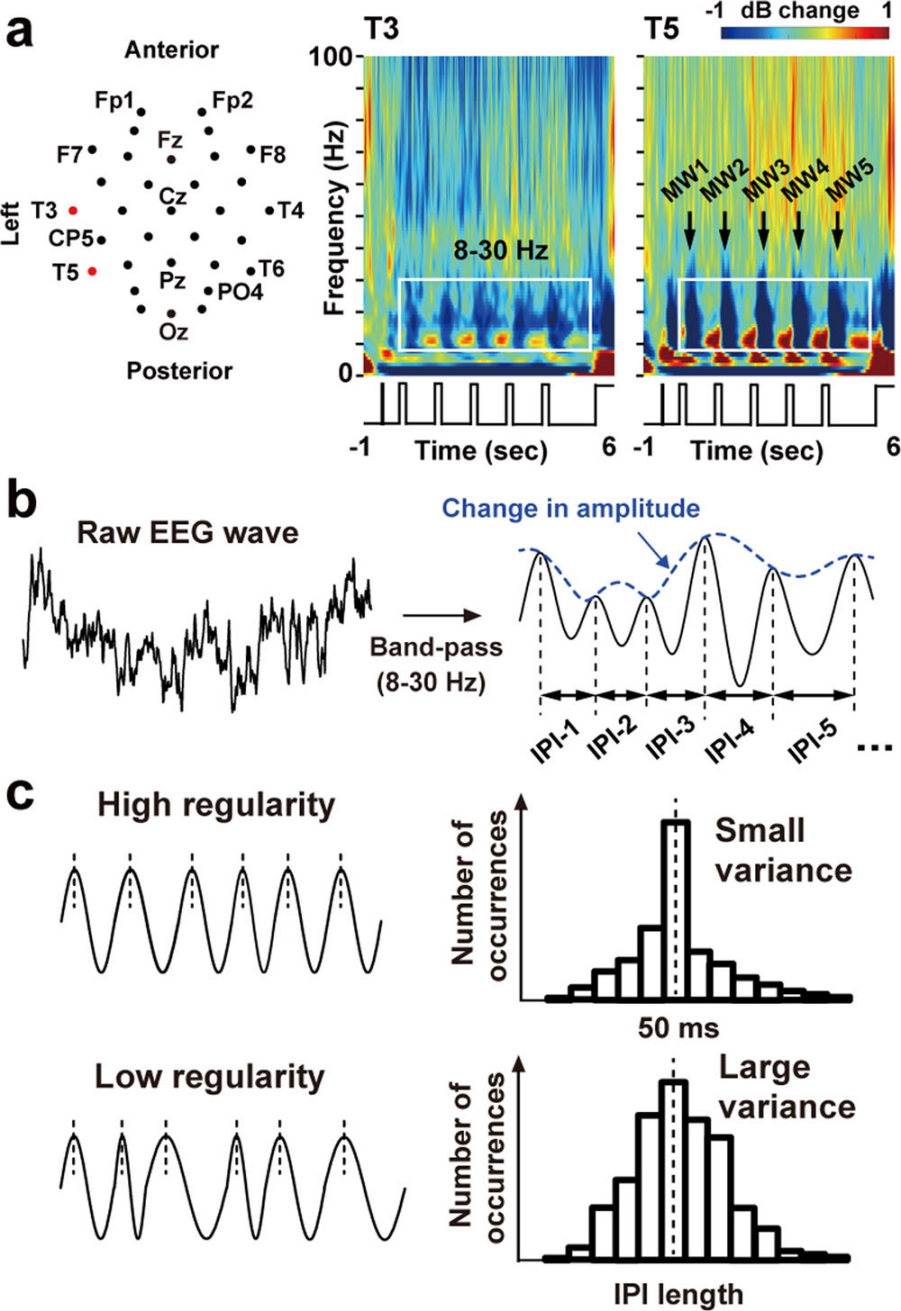
Measurements and analyses of electroencephalography (EEG) data. **a** Two-dimensional layout of 32 EEG sensors. Time-frequency power spectra (decibel power changes from a pre-cue period, −800 to −500 ms) at two sensors over the left temporal cortex (T3 and T5) are also shown. Prominent power changes throughout the five retention periods (D1–D5) are seen in alpha-to-beta band (8–30 Hz), indicated by a white rectangle. **b** Three measures of oscillatory signals. I first extracted waveforms at the alpha-to-beta band with a band-pass filter. Changes in amplitude were measured as an envelope of the filtered waveform (blue). Speed and regularity of the oscillatory signals were quantified as a mean and standard deviation (SD) of inter-peak intervals (IPIs), the time lengths between contiguous peaks of the filtered waveform. **c** Evaluation of oscillation regularity. Pooling all IPIs within a retention period (e.g., D5, 4300–5500 ms) generates a distribution of their occurrences as a function of IPI lengths (right panels). The higher regularity of oscillatory signals is indexed by a smaller variance or SD of the IPI distribution.

Neural activity was recorded from 32 points over the scalp. Figure 3a shows time-frequency power spectra over the left temporal cortex in SemR averaged across participants. Prominent power changes during the five retention periods (D1–D5) were observed in alpha-to-beta band (8–30 Hz). These data were consistent with mounting evidence from previous studies showing an involvement of alpha^24^, beta^21,25^, and alpha-to-beta^26–29^ rhythms in semantic processing and WM. I thus mainly focused on EEG signals in the alpha-to-beta band (8–30 Hz) below.

**Fig. 4:**
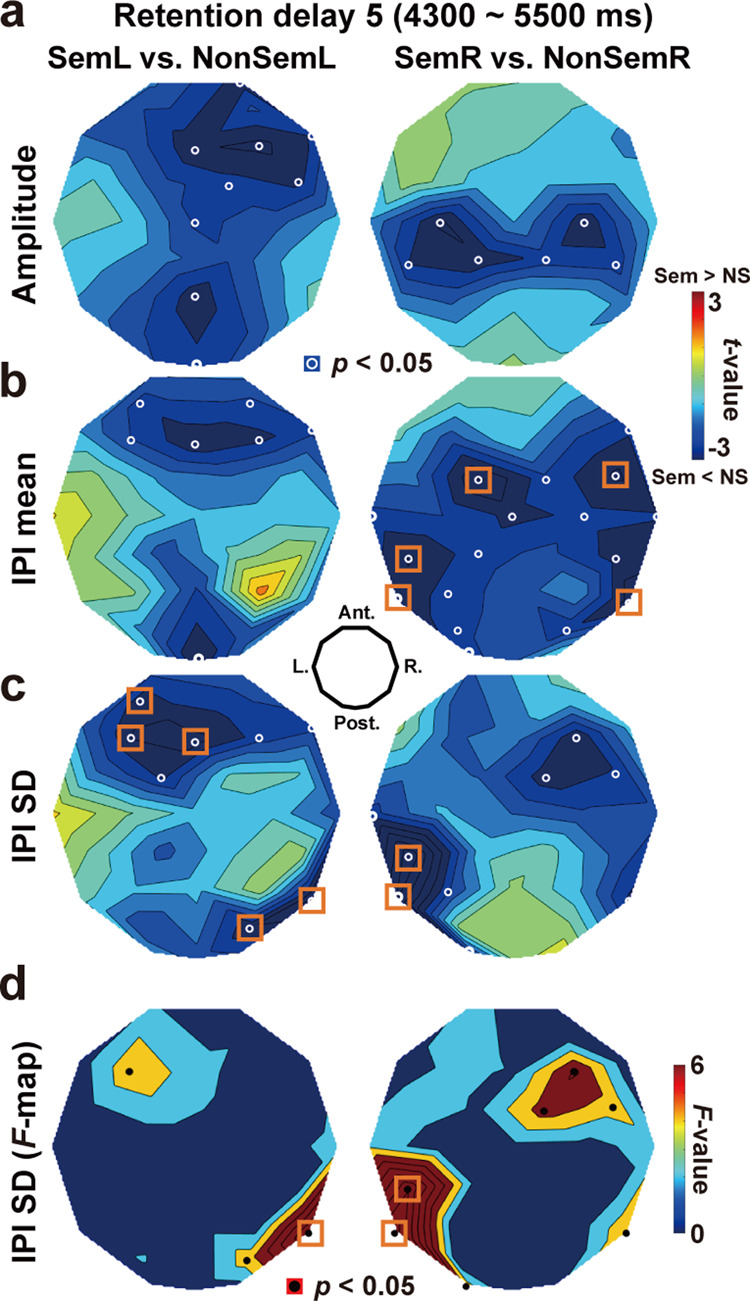
Effects of semantic relatedness on the three oscillatory measures. **a** *t*-map of oscillation amplitude (Fig. 3b, blue line). Mean amplitudes over 4300–5500 ms (delay 5) were compared between Sem and NonSem trials at each EEG sensor. Resultant *t*-values (negative: Sem < NonSem) were color-coded over the layout of 32 sensors. **b** *t*-map on mean IPIs **c** *t*-map on SDs of IPIs. White circles denote sensors showing a significant (*p* < 0.05, uncorrected) difference, while orange rectangles denote a significant difference after a correction of multiple comparisons. Memorizing 5 words semantically associated induced a reduction in SD of IPIs (EEG waveforms with higher regularity) over the temporal region contralateral to a cued hemifield (e.g., right temporal cortex in SemL). **d** *F*-map of IPI-SD. To exclude an effect of oscillation amplitude (panel **a**) as a possible confound, I performed ANCOVA (Sem vs. NonSem) with the difference in amplitudes included as a covariate. The reductions of IPI-SD (Sem < NonSem, indicated by positive *F*-values) were kept significant in the temporal regions.

Three different measures of oscillatory signals were analyzed; amplitude, speed, and regularity. Changes in the amplitude were measured as an envelope of the filtered waveform (dotted blue line in Fig. 3b). The speed and regularity of oscillatory signals were quantified by the mean and standard deviation (SD) of the inter-peak interval (IPI)^30^, respectively (Fig. 3c). Comparisons between Sem and NonSem trials during D5 are shown in *t*-maps depicted over the 2D layout of 32 sensors (Fig. 4). Since a paired *t*-test (Sem vs. NonSem) was repeated for 32 sensor positions, a problem of multiple comparisons was resolved by controlling false discovery rate (FDR). Although amplitudes of EEG waveforms at 8–30 Hz tended to be smaller in Sem than NonSem trials, no significant difference was observed after the FDR-correction (Fig. 4a). The *t*-map of mean IPI, a measure for oscillation speed, showed significant differences (Sem < NonSem) over frontal and temporal regions (orange rectangles in Fig. 4b). Although these data suggested an acceleration of brain rhythm related to a semantic integration, the change in mean IPI was limited to when participants memorized items in right visual field (SemR vs. NonSemR, right panel).

Finally, the SD of IPIs, a measure for irregularity of EEG waveforms, showed significant reductions in Sem compared to NonSem trials (Fig. 4c) both when memory items were presented in left and right visual fields. Clear differences were found over the posterior temporal cortex contralateral to a cued hemifield. A comparison of SemL vs. NonSemL indicated significant differences at electrode T6 (*t*(33) = −3.69, *p* = 0.0008, *d* = −0.09) and PO4 (*t*(33) = −2.95, *p* = 0.006, *d* = −0.09), while a comparison of SemR vs. NonSemR indicated significant differences at T5 (*t*(33) = −4.54, *p* = 0.00007, *d* = −0.15) and CP5 (*t*(33) = −4.92, *p* = 0.00002, *d* = −0.14). These results of IPI-SD were unchanged when I drew *F*-maps of ANCOVA (Fig. 4d) in which the difference in amplitudes (Fig. 4a) was included as a covariate. Semantic relatedness of MWs thus was associated with increased regularity (decrease in IPI-SD) of oscillatory signals. Indeed, the reduction in IPI-SD between Sem and NonSem was significantly correlated with the FA rates to lure probes (Supplementary Fig. 1), suggesting a close relationship of IPI-SD with a false memory (semantic integration).

### Changes in periodic and aperiodic components

**Fig. 5:**
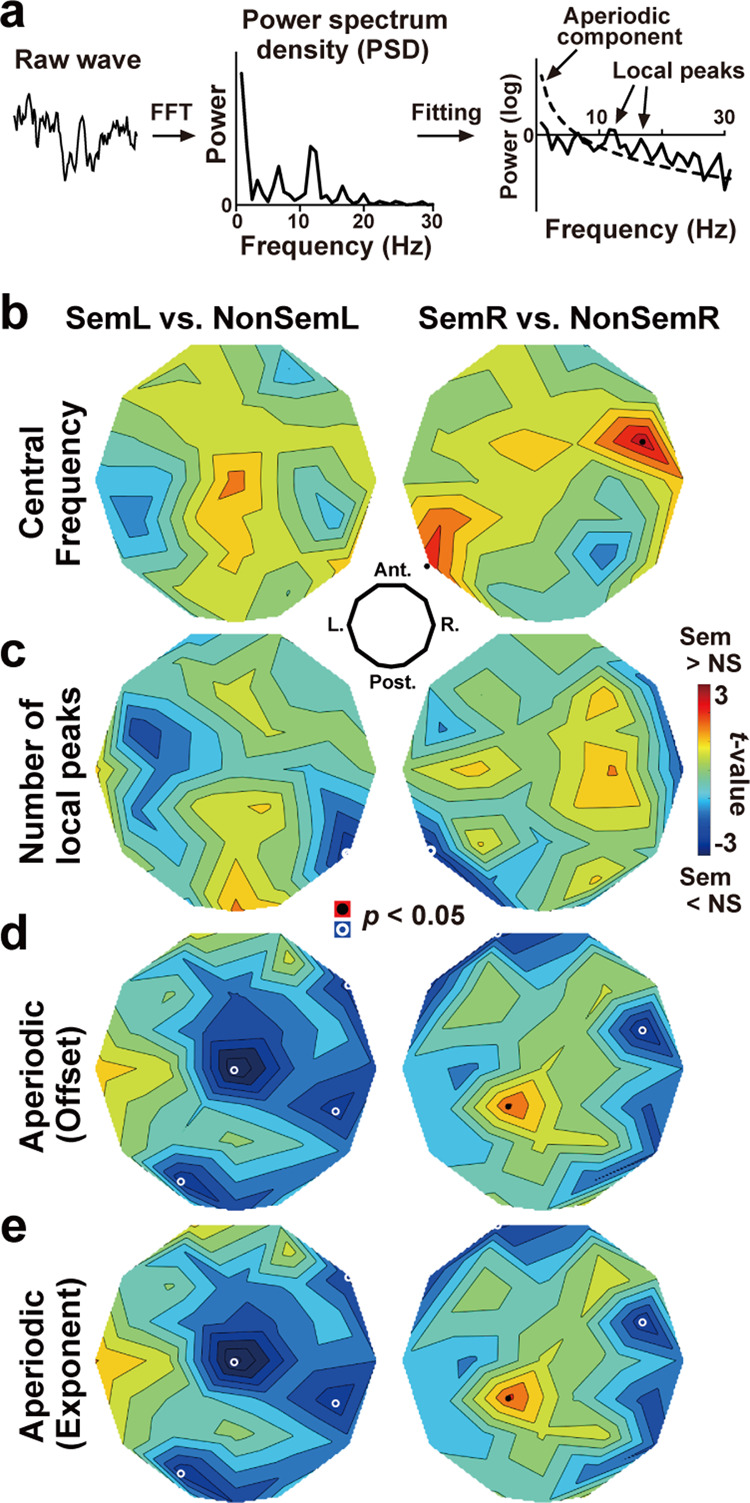
Analysis based on power spectrum density (PSD). **a** Procedures. A raw EEG waveform was converted to PSD. An aperiodic component of the PSD (dotted line) was modeled with an exponential function and separated from periodic components such as local peaks on the PSD. **b** *t*-maps of central frequency (a measure of oscillation speed) computed from the PSD at 8–30 Hz. A significant increase (SemR > NonSemR) was observed at the left temporal electrode (T5), which was consistent with mean IPI (Fig. 4b). **c** *t*-maps of a number of local peaks (a measure of irregularity). Significant decreases (Sem < NonSem) were observed at temporal regions contralateral to a cued hemifield, consistent with IPI-SD (Fig. 4c). **d** *t*-maps of an offset of the aperiodic component **e** *t*-map of an exponent of the aperiodic component.

Recent EEG studies have focused on a difference in periodic and aperiodic components of neural oscillatory signals on power spectrum density (PSD). A typical example is the FOOOF technique^31^ in which an aperiodic 1/*f* component was modeled and separated from periodic signals. I thus tested the validity of IPI analysis with the PSD-based approach. Using the fast Fourier transformation (FFT), a raw EEG waveform during D5 in each trial was converted into a PSD (Fig. 5a). An aperiodic (1/*f*) component was then estimated by fitting an exponential curve to the PSD at 8–30 Hz. Two parameters of the fitted curve, offset and exponent, were averaged across trials and compared between Sem and NonSem trials. I also computed two measures for a periodic component; central frequency and number of local peaks on the PSD. The central frequency represents the speed of oscillation of alpha-to-beta rhythm, while the number of peaks indexed its irregularity (see Fig. 1 and Methods for details).

Results are shown in Fig. 5b–e. An increase in central frequency was observed over the left posterior temporal cortex when participants retained MWs in the right visual field (SemR > NonSemR, T5 in the right panel of Fig. 5b: *t*(33) = 2.35, *p* = 0.025, *d* = 0.08). Significant decreases in the number of peaks (Sem < NonSem) were seen over the posterior temporal cortex contralateral to a cued hemifield (Fig. 5c, T6 in the left panel: *t*(33) = −2.53, *p* = 0.016, *d* = −0.50, T5 in the right panel: *t*(33) = −2.49, *p* = 0.018, *d* = −0.49). These results were overall consistent with the mean (Fig. 4b) and SD (Fig. 4c) of IPIs, respectively. On the other hand, no difference between Sem and NonSem was found in the aperiodic measures (offset and exponent, Fig. 5d and Fig. 5e) at the posterior temporal electrodes (T5 and T6, *p* > 0.23 for all). Semantic relatedness across the five MWs therefore induced changes in the periodic, rather than aperiodic, component of EEG signals.

### Time-related changes in the regularity of oscillatory signals

**Fig. 6:**
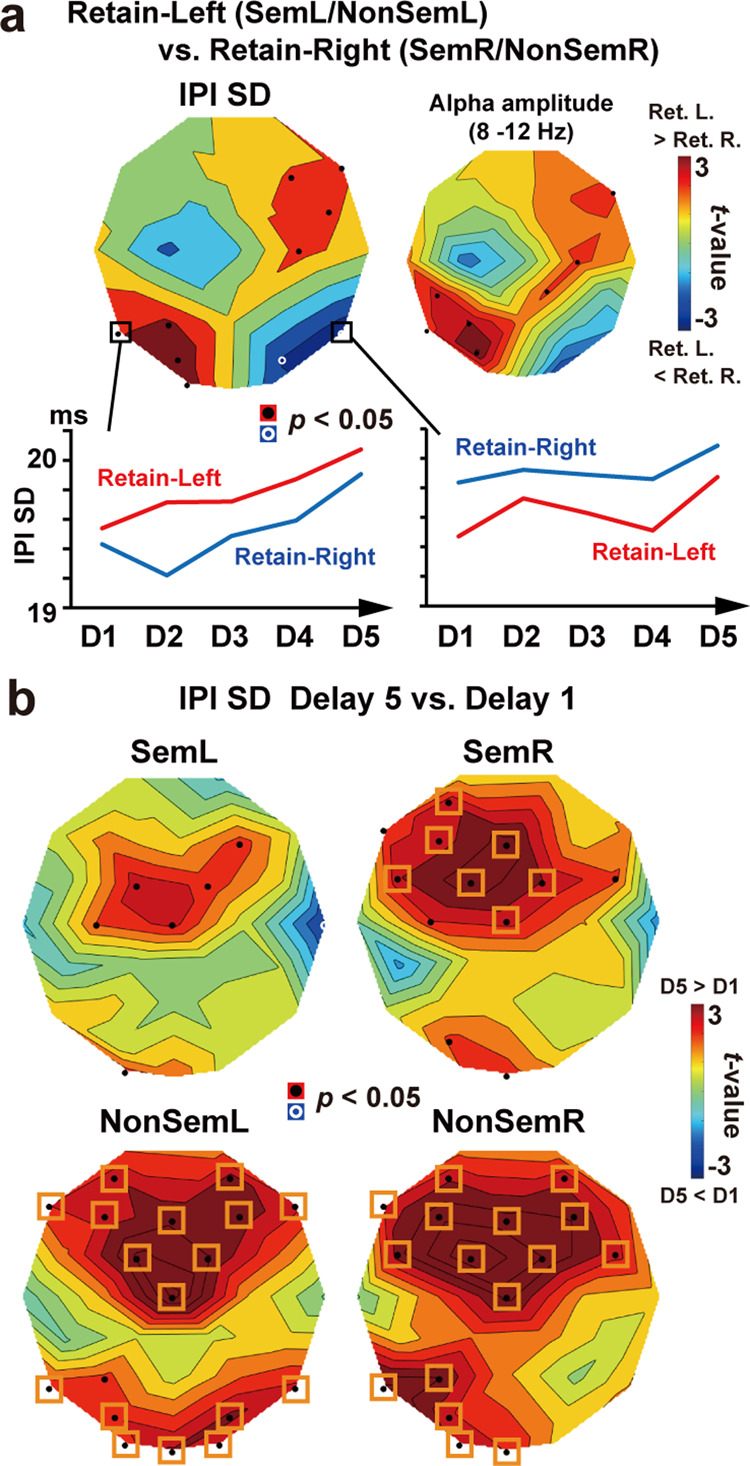
Changes in oscillation regularity over five delays (D1–D5). **a** *t*-map of IPI-SD (averaged across the 5 delays) between Retain-Left trials (SemL and NonSemL) and Retain-Right (SemR and NonSemR) trials. The same *t*-map for alpha amplitude (Retain-Left vs. Retain-Right) is also provided to confirm the attention-related alpha suppression reported in previous literature. Attentive processing of words in a left/right hemifield induces a decrease in SD (increase in regularity) over the right/left hemisphere. One can see, however, a gradual increase in SD from D1 to D5, which presumably reflects an accumulating memory load over time. **b** *t*-map of IPI-SD between D5 (4300–5500 ms) and D1 (300–1000 ms). In NonSem trials (lower panels), the time-related increase in SD (D5 > D1, shown in red) was prominent in the frontal cortex and posterior regions contralateral to a cued hemifield. This increase in SD was inhibited by semantic relatedness across MWs (Sem trials, upper panels), especially in the posterior regions. Black dots denote sensors showing a significant (*p* < 0.05, uncorrected) difference, while orange rectangles denote a significant difference after a correction of multiple comparisons.

More detailed information about SDs of IPIs is provided in Fig. 6. First, a comparison between Retain-Left trials (SemL and NonSemL) and Retain-Right trials (SemR and NonSemR) showed positive and negative *t*-values in the left and right occipito-temporal cortex, respectively (Fig. 6a). Positive *t*-values in the left cortex ranged from 2.93 (*p* = 0.006 at T5) to 3.87 (*p* = 0.0005 at PO3), while negative *t*-values in the right cortex ranged from −2.11 (*p* = 0.043 at PO4) to −2.82 (*p* = 0.008 at T6). Attentive processing of words in the left/right hemifield therefore induced a reduction of IPI-SD (increase in regularity) over the right/left hemisphere.

This neural signature of information processing (high regularity), however, diminished over time. One can see a gradual increase in IPI-SD from D1 (300–1000 ms) to D5 (4300–5500 ms), presumably reflecting an accumulating memory load. Figure 6b shows *t*-maps of IPI-SD between D1 and D5. In NonSem trials (lower panels), time-related increase in SD (D5 > D1, shown in red) was prominent in the posterior regions contralateral to a memory field (*t*(33) = 2.63, *p* = 0.013, *d* = 0.15 at T6 in NonSemL and *t*(33) = 4.13, *p* = 0.0002, *d* = 0.22 at T5 in NonSemR). These increases in SD were mitigated in Sem trials (upper panels, *t*(33) = 0.75, *p* = 0.46, *d* = 0.05 at T6 in SemL and *t*(33) = 0.67, *p* = 0.51, *d* = 0.04 at T5 in SemR), indicating that semantic integration of MWs inhibited a generation of irregular oscillatory waveforms in the posterior regions.

### Experiment 2

Results in Experiment 1 showed a higher regularity of EEG waveforms in Sem than NonSem conditions. This suggested that semantically related words induced similar patterns of neural oscillations that were easy to integrate when co-stored in vWM (Fig. 1a). I examined this point more directly in Experiment 2. Specifically, the same set of 300 words as Experiment 1 was presented individually (one by one) in Experiment 2. A semantic-correlation matrix for each pair of words (300 × 300) was compared with another correlation matrix (300 × 300) for neural oscillatory responses (IPIs) to those words. If those two matrices are highly similar to each other, this would show a link between semantic information and oscillatory responses, explaining the high-regularity signals in Sem trials of Experiment 1.

**Fig. 7:**
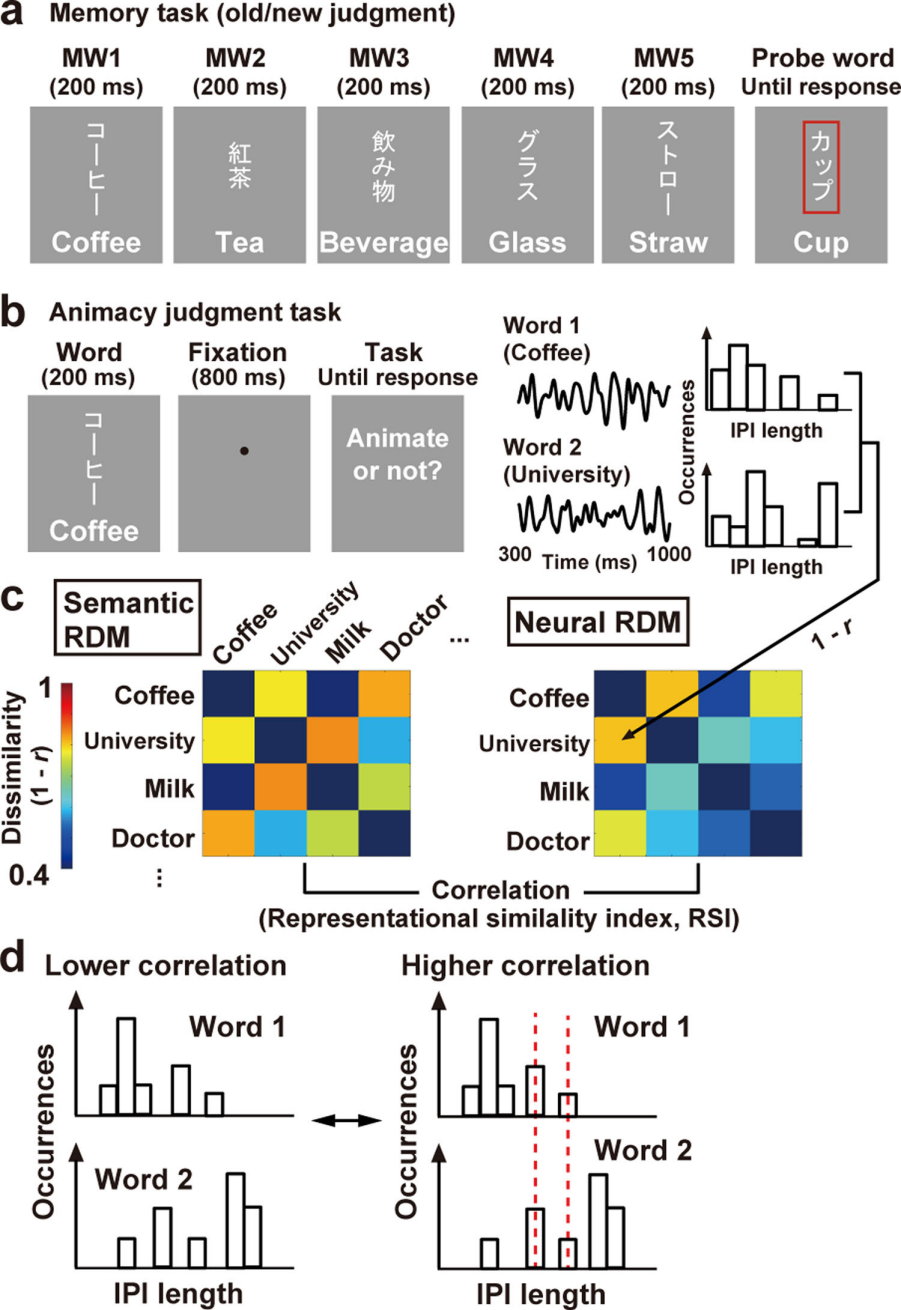
Experiment 2. **a** Memory task. Basic structures were the same as Experiment 1, except that each MW screen had only one word in its center. **b** Animacy judgment task. The same set of 300 words as the memory task was presented individually. Participants pressed one key to a word representing an animate object and pressed another to a non-animate object. **c** Representational similarity analysis (RSA). Using semantic vectors, I constructed a representational dissimilarity matrix (RDM, 300 × 300) showing a semantic distance (1 – *r*) for each pair of words (semantic RDM). I also made a neural RDM (300 × 300) based on a correlation of IPI histograms in response to each word in the animacy-judgment task. A high RSI (representational similarity index, a correlation between the semantic and neural RDMs) indicates that semantically associated words induce similar distributions of IPIs at that EEG sensor. **d** The correlation of IPI histograms between two words. EEG waveforms in alpha-to-beta band (8–30 Hz) produced about 15 IPIs in a time window of analysis (300–1000 ms after a word onset). A higher correlation would be observed when two histograms exhibit overlapping changes in the speed of neural oscillations.

Each participant in Experiment 2 performed two tasks (Fig. 7). The first task involved a memory of five words sequentially presented (Fig. 7a). This was identical to the vWM task in Experiment 1, except that there was no attentional direction by the cue (a MW screen in Experiment 2 had only one word in its center). In the second (main) task, the same set of 300 words as Experiment 1 was presented one by one (Fig. 7b). Participants performed an animacy judgment task on each word, pressing one key to animate and another to non-animate objects.

### Results of the memory task

**Fig. 8:**
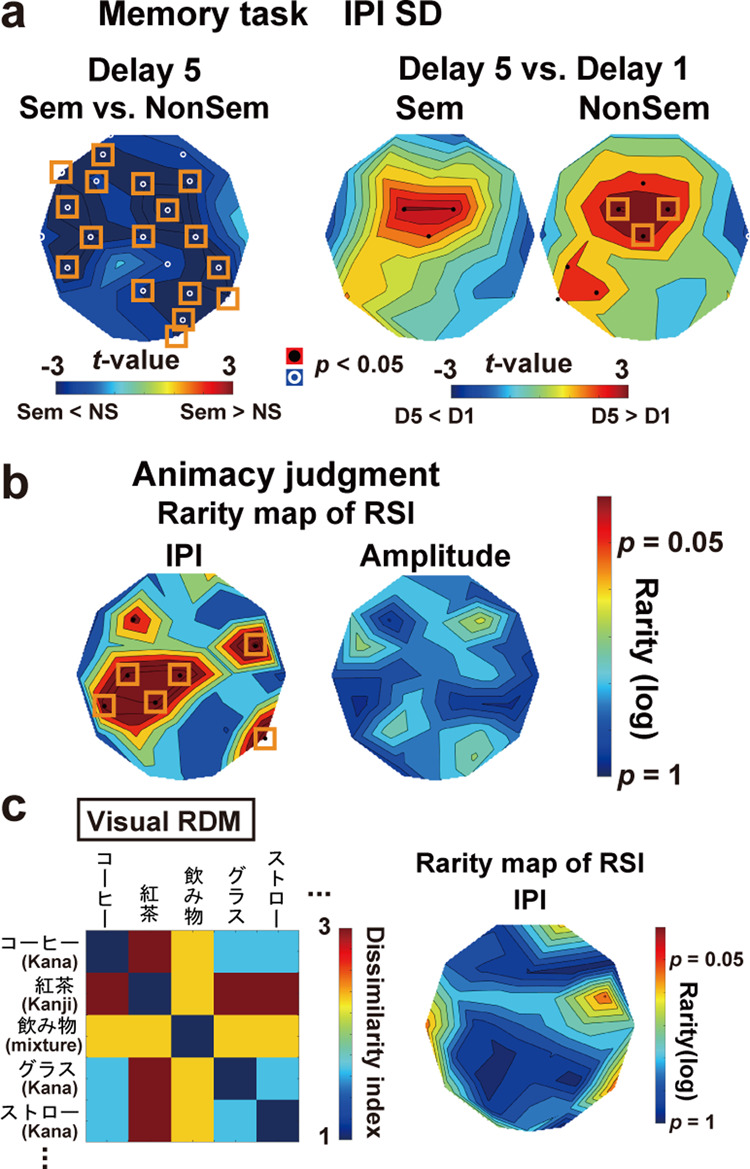
Results of Experiment 2. **a** Memory task. The SD of IPIs during D5 was found to be lower in Sem than NonSem trials (left panel). Semantic associations across five MWs in Sem trials mitigated a time-related increase in SD (D1 < D5) over the left temporal regions (right panels). Those results replicated Experiment 1. **b** A rarity map of RSI in animacy judgment task. The rarity (*p*-value) of RSI in actual data was estimated through a random permutation of semantic RDM for 1000 times (see text for details). Significant RSIs (corrected for multiple comparisons, orange rectangles) were observed when a neural RDM was made from the correlation of IPI histograms (left), indicating that semantically-associated words induced similar sets of IPIs. No significant RSI was observed when neural RDM was made from the correlation of amplitude changes (not IPIs) between two words (right). **c** Control. A new RDM based on visual similarity between two words (visual RDM, left panel) was compared with neural RDM in Fig. 7c. No significant RSI was observed in the rarity map (left panel), indicating that correlations of IPI histogram were not modulated by visual factors.

Means and SEs of *d’* in the memory task in Experiment 2 were 3.56 ± 0.09 in Sem and 3.74 ± 0.12 in NonSem trials. No significant difference was observed (*t*(26) = 2.01, *p* = 0.055, *d* = 0.32). The FA rate to lure probes (5.56 ± 1.72%) was significantly higher (*t*(26) = 2.51, *p* = 0.019, *d* = 0.55) than that to new probes (1.85 ± 0.66%). These behavioral results were similar to those in the Retain-Right trials in Experiment 1. The *t*-maps of IPI-SD are shown in Fig. 8a. Oscillatory signals during D5 were more regular in Sem than NonSem trials (left panel. The most distinct difference over the left temporal region was seen at electrode CP5 (*t*(26) = 3.33, *p* = 0.003, *d* = 0.15). The time-related increase in IPI-SD (from D1 to D5) was inhibited in Sem trials (middle panel. *t*(26) = 1.21, *p* = 0.24, *d* = 0.06 at CP5) but not in NonSem trials (right panel. *t*(26) = 2.25, *p* = 0.033, *d* = 0.15 at CP5). Those data replicated Experiment 1.

### Results of the animacy judgment task

EEG data in the animacy judgment task were used for the representational similarity analysis or RSA^32–34^. First, I made a representational dissimilarity matrix (RDM) reflecting a semantic distance for each pair of the 300 words (Fig. 7c), using the word vectors on fastText library (https://fasttext.cc/). Next, I made another RDM based on a correlation of IPIs (neural RDM). A histogram of alpha-to-beta IPIs at 300–1000 ms was depicted for each word. Each cell (dissimilarity index or DI) of the neural RDM was defined as 1 − *r*, where *r* was a correlation of IPI histograms between two words. As shown in Fig. 7d, a higher correlation would be observed when two histograms exhibit overlapping changes in the speed of neural oscillations. Finally, a correlation between semantic RDM and neural RDM was computed at each EEG sensor (representational similarity index or RSI). A high RSI indicates that semantically associated words produced similar distributions of IPIs. The statistical significance of those RSI was evaluated through a comparison with random data.

The mean and SE of accuracy in the animacy judgment task was 99.28 ± 0.28%. A rarity map of RSI between the semantic and neural RDMs is shown in the left panel of Fig. 8b. Significant RSIs were found in the frontal, parietal, and temporal regions, especially in the left hemisphere. Six electrodes with significant *p*-values after the FDR corrections were as follows; CP5 (*p* = 0.009), C3 (*p* = 0.007), CP1 (*p* = 0.008), Cz (*p* = 0.005), FC6 (*p* = 0.003), T6 (*p* = 0.004). In Supplementary Fig. 2, I provided results of RSA separately conducted for 34 words representing animate objects and 266 words representing non-animate objects. The RSI over the left temporal regions was significant in all analyses. These data showed that semantically associated words induced similar sets of IPIs, which were consistent with the high regularity of oscillatory signals when co-stored in vWM (Fig. 8a). In contrast, no significant RSI was observed when the semantic RDM was compared with the neural RDM made from the correlation of amplitude changes (envelopes of 8–30 Hz waveforms at 300–1000 ms) between two words (uncorrected *p* > 0.106 for all, right panel of Fig. 8b). Semantic information was thus represented in IPIs, not in amplitude, of oscillatory signals.

As a control, I performed another RSA using an RDM reflecting visual (not semantic) similarities of the 300 words (Fig. 8c, see Methods for details). Non-significant RSI in this analysis (uncorrected *p* > 0.046 for all, right panel) indicated that visual factors had no effect on the distribution of alpha-to-beta IPIs at 300–1000 ms.

## Discussion

In the present study, I compared neural oscillatory signals when human participants retained the information of five words semantically related (Sem trial) or not (NonSem trial). Results revealed a reduced SD (increased regularity) of IPIs in Sem than NonSem conditions over the temporal cortex contralateral to memory items (Exp.1). The reduction of mean IPIs (an acceleration of brain rhythm) was also observed when memory items were presented in the right visual field (left hemisphere). In Experiment 2, I presented the same set of words individually in a non-memory (animacy judgment) task, finding that semantically related words induced similar distributions of IPIs. These results suggested that memorizing words with a common semantic feature would induce a resonance (or sharing) of the oscillatory signals across the words, resulting in increased regularity of EEG waveforms during the retention period (Fig. 1).

In both Experiments 1 and 2, key regions for semantic integration were found over the posterior temporal lobe. This is consistent with recent evidence. For example, Volfart et al. (2021) reported neural activity in the posterior temporal cortex related to a semantic categorization (e.g., discriminating animals from city names) of visually presented words^35^. In light of previous literature, a source region for present data might be the posterior middle temporal gyrus (pMTG)^36,37^. In order to integrate the information of five words, the brain had to access semantic knowledge of each word and connect the words under a common concept. The pMTG is thought to play a critical role in such a process involving an immediate recall and re-structuring of semantic networks^38^.

Several previous studies have investigated the effect of across-word (or across-object) relationships on oscillatory signals in the human brain^27^. They, however, mainly analyzed changes in oscillatory powers, such as an alpha-power decrease during the retention of semantically related words^39^ and a beta-power decrease induced by the sentence superiority effect^40^. Consistent with those data, I found decreases in the power of alpha-to-beta rhythm in Sem compared to NonSem trials (Fig. 4a). Replicating those previous findings, I further showed that semantic relatedness of words also modulated temporal measures (speed and regularity) of brain rhythms.

Previous studies have proposed cross-frequency couplings as a neural model of multi-unit memory. A typical example is the phase-amplitude coupling between theta and gamma rhythms^41–43^. In this model, individual memory items are represented by oscillatory signals in a gamma range. Information of multiple items is retained by sequentially activating their representations (gamma activities) on a theta cycle. Although this model has been supported by compelling evidence^44–50^, it does not mention explicitly how the information of items is integrated into the buffer of WM. Indeed, this line of studies has focused on how the brain segregates (not integrates) information of semantically related concepts, e.g., by reactivating them in distant phases of theta cycle^51,52^. The present data would make up for this point by suggesting a new model of semantic integration through the sharing of oscillatory signals across memory items (Fig. 1). One possibility is that the brain retains the information of multiple items by two different mechanisms. The mechanism of theta-gamma coupling encodes a sequence of individual items by keeping their independence, while the alpha-to-beta rhythm represents the semantic relatedness of those items and enables an efficient encoding such as chunking.

On the other hand, the present study has several limitations described below. First, the model in Fig. 1 might be oversimplified, given that neural underpinnings of semantic information have been mostly unclear and controversial^53^. Although I illustrated in Fig. 1 that each feature was retained as the oscillation of a specific frequency, the present results are also consistent with more complex models, for example, in which a single feature is recalled as combined signals of multiple frequencies. Second, the present data should be interpreted cautiously in light of a separation between vWM and mental lexicon (long-term memory). Although changes in IPI measures in Experiment 1 were related to semantic integration across words in vWM, the present study did not investigate directly the semantic knowledge (long-term memory) stored in the mental lexicon. Further studies are needed to analyze relationships between neural oscillatory signals and long-term (semantic) memory. Indeed, previous studies showed a separation of brain regions storing semantic knowledge from regions for vWM. The anterior temporal lobe is known as the semantic “hub” in which various conceptual knowledge is encoded as the long-term memory^38,54^. In contrast, posterior regions such as the pMTG were related to control of WM contents^55^, typically activated by a semantic integration across words. As discussed above, the present results (reduction of IPI-SD) were mainly seen over the posterior regions (Fig. 4) and thus would reflect the WM process.

In conclusion, the present data provided insight into how verbal information was integrated as neural (electric) signals in the healthy human brain. Semantic chunking is known to be a key method to enhance one’s memory capacity. The current data might be also useful to develop a new method to prevent age-related degradation of vWM^56^.

## Methods

### Participants

Thirty-four healthy subjects (native speakers of Japanese) participated in Experiment 1 (17 females, age range: 18–42). This sample size (34) was determined by a power analysis using G*Power 3^57^. The type I error rate and statistical power were set at 0.05 and 0.80, respectively. Effect size was assumed to be middle (0.5)^58^ because I found no previous study having the same goal as the present one. Data of one subject (female) were excluded from analysis due to excessive noise in EEG waveforms and thus replaced by data of an additional participant (female). Laterality quotients (LQs) measured by the Edinburgh Handedness Inventory^59^ showed that all participants were right-handed (mean: 84.76, range: 11.11–100) but two (−11.11 and −17.65). Thirty healthy subjects participated in Experiment 2. Data of three participants were excluded from analyses because of a technical problem (loss of EEG data, *N* = 1) and excessive noise (*N* = 2), resulting in 27 participants in a final dataset (11 females, mean LQ: 80.28). All participants had normal or corrected-to-normal visual acuity. After the nature of the study had been explained, I received informed consent from each participant. All experiments were conducted in accordance with regulations and guidelines approved by the ethics committee of Kobe University, Hyogo, Japan.

### Task (Experiment 1)

All visual stimuli were generated with the Matlab Psychophysics Toolbox^60,61^ and presented on a CRT monitor (refresh rate: 60 Hz). Each trial started with a black fixation point (0.18 × 0.18 deg) over a gray background for 800 ms. This was followed by a cue stimulus (an arrow pointing leftward or rightward, length: 1.34 deg, duration: 33 ms) over a central field to direct the attention of participants (Fig. 2a). After another fixation period (467 ms), participants viewed two Japanese words (nouns), one in left and another in right visual fields, presented simultaneously for 200 ms (memory-word screen). Those words consisted of 1–5 white Japanese letters (Kana and Kanji characters) vertically arranged. The size of each letter was 1 (H) × 1 (V) deg, and the center-to-center distance between the fixation point and word was 1.25 deg. A total of five screens (ten words) were sequentially presented with an inter-screen interval (delay) of 800 ms. After the last (5th) delay of 1300 ms, the trial ended with a probe word (marked by a red rectangle, always shown in the cued visual field). Participants were asked to memorize five words in the cued hemifield (memory words or MWs) and performed an old/new judgment on the probe word. They pressed one button if the probe matched any of the five words they retained (“old” response) but pressed another if not (“new” response). They were also instructed to ignore five words presented in an uncued hemifield. The ten words shown in one trial were different from each other (i.e., no word appeared both in cued and uncued hemifields in the same trial). No time limitation was imposed for this old/new judgment.

An effect of semantic integration on EEG waveforms was measured by a comparison of Sem and NonSem trials. In Sem trials, five MWs in the cued hemifield were semantically related (e.g., “piano”, “band”, “melody”, “concert”, and “jazz”), while they were not in NonSem condition (e.g., “curtain”, “jazz”, “rose”, “pencil”, and “kitchen”). Importantly, words in both conditions were taken from the same list of 300 words so that total visual inputs across all trials were balanced between Sem and NonSem. A combination of cued hemifield (left/right) and semantic relatedness (Sem/NonSem) produced four types of trials. Trials with a leftward cue and related MWs were called as SemL, while those with a rightward cue and unrelated MWs were called as NonSemR. An experimental session contained 60 trials in which those four types of trials (15 for each) were intermixed in a random order. The whole experiment consisted of four sessions.

Each of the four conditions (SemL, SemR, NonSemL, and NonSemR) had 60 trials in total. In SemL and SemR, one of five MWs was shown as a probe in 20 out of the 60 trials (old-probe trial, e.g., “jazz” in the above case). Serial positions (1 - 5) of the probe word were balanced (four trials for each position) to confirm the primacy and recency effects. A probe word not included in MWs was shown in another 20 trials (new-probe trial, e.g., “steak”). The new probe was either taken from five words in the uncued hemifield in the same trial (unattended new probe, 10 trials) or totally new (unseen new probe, 10 trials). In the other 20 trials, I showed a probe that was semantically related to the MWs but had never appeared (lure probe, e.g., “rhythm”). In NonSemL and NonSemR, 30 trials had an old probe, and the other 30 trials had a new probe (unattended new probe in 20 trials and unseen new probe in 10 trials).

### Analysis of behavioral data

Behavioral data were analyzed using the signal detection theory. For each of the four conditions, I computed a hit rate in which participants answered “old” to an old probe and a false-alarm (FA) rate in which they answered “old” to a new probe. A measure of sensitivity (*d’*) was computed^62^ using the equation

$$d{\rm{\mbox{'}}}=z({\rm{hit}\,{rate}})-z({\rm{FA}\,{rate}})$$

where *z* denotes the inverse cumulative normal function. If semantic relatedness across MWs facilitated the old/new judgment, this would be observed as higher *d’* in Sem than NonSem conditions.

Semantic integration, however, is also known to exert a negative influence when the probe is related to memory words (false memory). I examined this point by comparing the FA rates between the lures and new (unseen) probes of the Sem condition. In the case of the lure probe, the FA rate was defined as a percentage of trials in which participants answered “old” to a lure probe. Since each condition had 20 trials with lure probes, an individual FA rate ranged from 0 to 100% in a step of 5%. False memory would be indexed by a higher FA rate to lures than that to new probes.

### Stimuli (words)

MWs in each trial were taken from a list of 300 Japanese nouns prepared for the present study. Most words in this list were made by translating English words in the Deese–Roediger–McDermott (DRM) list^63^ into Japanese, although some words were arranged with procedures of Miyaji and Yama^64^. A list of five MWs in the Sem trial was determined based on the DRM list (e.g., “piano”, “band”, “melody”, “concert”, and “jazz”), while a list in NonSem trial was made by choosing five words unrelated to each other. Each word was used twice per condition to generate 240 trials in total. Specifically, a given word was shown eight times throughout the experiment; twice in SemL (at cued hemifield in one trial and at uncued hemifield in another trial), twice in SemR, twice in NonSemL, and twice in NonSemR.

The validity of those lists was checked by computing semantic correlations among five MWs. I obtained a semantic vector (1 × 300) for each of the 300 words from the fastText library (https://fasttext.cc/). Semantic relatedness between two MWs was measured as a correlation coefficient of those vectors. As shown in Fig. 2b, semantic correlations among five MWs (averaged across all trials) were 0.39 – 0.42 in Sem and 0.18–0.22 in NonSem. I also confirmed that correlations between MWs and lure probes (0.41–0.45) were higher than those between MWs and new probes (0.19–0.20).

Other linguistic factors, such as phonological variations, were also controlled. I measured a within-trial variation of phonological factors by analyzing the Japanese mora of each word^65^. For each trial, a total number of moras used over five MWs was computed, with a mora shared by more than two MWs counted as one (phonological variation). No significant difference was observed (*t*(238) = 0.42, *p* = 0.67, Cohen’s *d* = 0.05) between Sem (12.87 moras) and NonSem trials (12.98 moras).

### EEG measurements

Neural activity was recorded with the ActiveTwo system by Biosemi (Amsterdam, Netherlands). I measured EEG signals at 32 points over the scalp; FP1, FP2, AF3, AF4, F7, F3, Fz, F4, F8, FC5, FC1, FC2, FC6, T7, C3, Cz, C4, T8, CP5, CP1, CP2, CP6, P7, P3, Pz, P4, P8, PO3, PO4, O1, Oz, and O2 (Fig. 3a). Data were recorded with a sampling rate at 2,048 Hz and an analog low-pass filter of 417 Hz. Offsets of all electrodes were kept from −20 to 20 mV. Preprocessing of EEG data was performed with the Brainstorm toolbox^66^ for Matlab. I first removed a fixed-frequency noise from the power line (60, 120, and 180 Hz) using a notch filter. A band-pass filter of 0.1–200 Hz was also applied to eliminate low- and high-frequency noises. For the data of six participants, a band-pass filter of 0.5–200 Hz was used because their data contained much low-frequency noise, presumably caused by body movements. Results when the filter of 0.5–200 Hz was applied to all 34 participants are shown in Supplementary Fig. 3. All data were then referenced with an average potential over the 32 electrodes. I segmented EEG waveforms into each trial (epoch range: −1300 to 7000 ms relative to the onset of the 1st MW screen) and classified them into the 4 conditions. Waveforms with a max-min amplitude larger than 150 μV at -700 to 5500 ms were excluded from analyses. The numbers of trials that remained after the rejection (mean ± SD) were 44.15 ± 12.79 (SemL), 42.97 ± 13.03 (SemR), 44.59 ± 12.01 (NonSemL), and 43.85 ± 12.47 (NonSemR). A two-way ANOVA of Sem/NonSem × L/R indicated no main effect or interaction (*F*(1,33) < 2.77, *p* > 0.10, *η*^2^ < 0.078 for all).

### Time-frequency power analysis

I first performed a time-frequency analysis to get an overview of power changes in oscillatory signals (Fig. 3a). Following the decomposition procedures with complex Morlet wavelets, the segmented EEG waveform was converted into a power spectrum of time (-1300 to 7000 ms, resolution: 512 Hz) × frequency (1 to 100 Hz, resolution: 1 Hz). The central frequency and time resolution (at full width at half maximum) of the Morlet wavelet were set at 1 Hz and 3 s, respectively. The spectrum was then averaged across all trials in each condition. Finally, I performed a baseline correction^67^, converting all data (−1300 to 7000 m) into decibel change from a pre-cue period (-800 to -500 ms).

### Inter-peak interval (IPI) analysis

I then analyzed three different measures of oscillatory signals (Fig. 3b); amplitude, speed, and regularity. The speed and regularity are oscillatory measures used in recent studies on WM^31,68–70^. Using the Hilbert transformation, I measured changes in amplitude as an envelope of the filtered waveform in a frequency band of interest (dotted blue line in Fig. 3b). On the other hand, the speed and regularity of oscillatory signals were quantified by the inter-peak interval (IPI) analysis^30^. Using a Matlab function (findpeaks.m), I first identified all peaks on the filtered EEG waveform of each trial. The IPIs were measured as time lengths between contiguous peaks. A mean length of IPIs pooled over a given period (e.g., 4300–5500 ms in case of delay 5) and across all trials indexes a speed of neural oscillations because slow/fast oscillatory signals produce longer/shorter IPIs. The regularity of neural waveforms, in contrast, was measured as a standard deviation (SD) of the pooled IPIs. As shown in Fig. 3c, irregular neural oscillations are characterized by a larger variance of IPIs, because such waveforms produce IPIs distant from the mean.

### Statistical procedures

An effect of semantic relatedness across MWs on neural activity was investigated by a comparison of Sem and NonSem trials. Of particular interest was the data in delay 5 (D5), where a memory load became maximum. Avoiding visually-evoked potentials in response to the 5th MW screen (4000–4200 ms), I compared the three measures of oscillatory signals at 4300–5500 ms between Sem and NonSem (Fig. 4). This analysis window starting from 300 ms after the 5th MW screen was consistent with previous EEG studies on WM^71,72^ and semantic integration^73,74^. Since a paired *t*-test (within-subject comparison, two-sided) was repeated for 32 sensor positions, the problem of multiple comparisons was resolved by controlling the false discovery rate (FDR). I adjusted a statistical threshold based on the Benjamini–Hochberg correction^75^ with the *q*-value set at 0.05. Sensors showing a significant difference after this correction were marked with orange rectangles in Fig. 4 and Fig. 6.

### Analysis of power-spectrum density (PSD)

An increasing number of studies focused on a difference in periodic and aperiodic components of neural oscillatory signals^31,76^. A typical approach is the FOOOF (fitting oscillations and one over *f*) in which an aperiodic 1/*f* component of power spectrum density (PSD) is separated from periodic components through the fitting with an exponential curve^31^. I applied this FOOOF analysis to the present data in order to validate the results of the IPI analysis.

First, an EEG waveform at 4300–5500 ms in each trial was converted into PSD with the fast Fourier transformation (FFT, Fig. 5a). Using the code of FOOOF available online (https://fooof-tools.github.io/fooof/), I then estimated an aperiodic component of the PSD at 8–30 Hz and separate it from a periodic component. Default settings were used for this estimation (peak_width_limits = [0.5, 12], max_n_peaks = ‘Inf’, min_peak_height = 0.0, peak_threshold = 2.0, aperiodic_mode = ‘fixed’). Based on the results of FFT and FOOOF, I computed four PSD-based measures; central frequency, number of local peaks on PSD, offset of aperiodic component, and exponent of aperiodic component. They were averaged across all trials and compared between Sem and NonSem trials (Fig. 5b–e)

The central frequency was the weighted mean of frequencies × powers^69,77,78^ and indexed a speed of neural oscillation. The number of peaks at 8–30 Hz was a measure of periodic signals representing neural irregularity. If the semantic integration was associated with a harmonic oscillatory signal, a smaller number of peaks would be observed in Sem than in NonSem trials (Fig. 1). On the other hand, if the results of IPI analysis (Fig. 4) reflected changes in an aperiodic, rather than periodic, component of EEG signals, a difference in the offset or exponent would be observed between Sem and NonSem trials.

### Experiment 2

Results in Experiment 1 showed a higher regularity of EEG waveforms in Sem than NonSem conditions. This suggested that semantically related words induced similar patterns of neural oscillations that were easy to integrate when co-stored in vWM (Fig. 1a). I examined this point more directly in Experiment 2. Specifically, the same set of 300 words as Experiment 1 was presented individually (one by one) in Experiment 2. A semantic-correlation matrix for each pair of words (300 × 300) was compared with another correlation matrix (300 × 300) for neural oscillatory responses to those words. If those two matrices are highly similar to each other, this would show a link between semantic information and oscillatory responses, explaining the high-regularity signals in Sem trials of Experiment 1.

Each participant in Experiment 2 performed two tasks (Fig. 7). The first task involved a memory of five words sequentially presented (Fig. 7a). This was identical to the vWM task in Experiment 1, except that there was no attentional direction by the cue (a MW screen in Experiment 2 had only one word in its center). Participants underwent 2 sessions of 60 trials in which Sem and NonSem trials were intermixed in a random order. There were three types of probes in the Sem condition (20 trials with old probes, 20 trials with new probes, and 20 trials with lure probes) but two types of probes in the NonSem condition (30 trials with old probes and 30 trials with new probes). In the second (main) task, the same set of 300 words as Experiment 1 was presented one by one (Fig. 7b). Participants performed an animacy judgment task on each word, pressing one key to animate and another to non-animate objects (150 trials × 2 sessions). An experiment started with the animacy judgment task, followed by the memory task. Other details (measurements of EEG data and analyses of behavioral data) were identical to Experiment 1.

### Representational similarity analysis

EEG data in the animacy judgment task were used for the representational similarity analysis or RSA^32–34^. First, I made a representational dissimilarity matrix (RDM) reflecting a semantic distance for each pair of 300 words (Fig. 7c). Each cell in this semantic RDM (dissimilarity index or DI) was defined as 1 − *r*, where *r* was a correlation between semantic vectors of two words (obtained from the fastText library, https://fasttext.cc/). Next, I made another RDM based on a correlation of IPIs (neural RDM). A histogram of alpha-to-beta IPIs at 300–1000 ms was depicted for each word, with its vector defined as

$${\rm{V}}_{\rm{A}}=[{\rm{X}}_{1}{\rm{X}}_{2}\ldots {{\rm{X}}}_{2048}]$$

where V_A_ denotes an IPI vector of the word A, and X_1_–X_2048_ shows numbers of IPIs at bin 1 (0.488 ms) to bin 2048 (1000 ms). Each DI in the neural RDM was 1- *r*, where *r* was a correlation between IPI histogram vectors of two words (e.g., V_A_ vs. V_B_). As shown in Fig. 7d, a higher correlation would be observed when two histograms exhibit overlapping changes in speed of neural oscillations

Finally, a correlation between semantic and neural RDMs was computed at each EEG sensor (representational similarity index or RSI). Since bottom-left DIs were identical to top-right DIs in each RDM, I compared the bottom-left halves of the semantic and neural RDMs, excluding diagonal components (0). A high RSI indicates that semantically associated words produced similar distributions of IPIs. The statistical significance of those RSI was evaluated through a comparison with random data. By permutating the word labels of semantic RDM randomly for 1000 times, I generated a distribution of RSIs under a hypothesis of null effect. The rarity (*p*-value) of RSI in actual data was estimated as its percentile in this null distribution. A problem of multiple comparisons over the 32 sensors was resolved by the FDR-controlling approach in Experiment 1.

As a control, I tested whether distributions of alpha-to-beta IPIs were modulated by visual (not semantic) factors of word stimuli. A new RDM reflecting visual dissimilarities of word pairs was made for this analysis (visual RDM, Fig. 8c). The 300 words in the present study consisted of two types of Japanese letters: Kana (phonograms) and Kanji (ideograms, imported from China). The Kanji letters are characterized by higher spatial frequency and complexity than Kana letters^79^. I thus classified the 300 words into three categories; (i) Kana words, (ii) Kanji words, and (iii) mixtures of Kana and Kanji. The DI in the visual RDM was set at 1 when two words belonged to the same category and at 3 when one was a Kana word, and the other was a Kanji word. A DI of 2 was given to a word pair of a Kana–Kanji mixture and a Kana/Kanji word. A rarity map of RSIs between the visual and neural RDMs was then computed and shown as the right panel in Fig. 8c.

### Reporting summary

Further information on research design is available in the Nature Research Reporting Summary linked to this article.

### Supplementary information

Supplementary Figures 1-3

Reporting summary

## Data Availability

The data that support the findings of this study are available at 10.7910/DVN/R2DBYS.

## Code availability

Matlab codes written for this study are available from the corresponding author (Y.N.) upon request for academic purposes
