# Supplementary material for: Harmonic memory signals in the human cerebral cortex induced by semantic relatedness of words
Source: NPJ Sci Learn. 2024 Feb 14;9:6. doi: 10.1038/s41539-024-00221-1 (PMC10866900; doi:10.1038/s41539-024-00221-1)
Supplement: Supplementary file 1 — Supplementary Figures 1-3 [file 41539_2024_221_MOESM1_ESM.pdf]

Supplementary Information

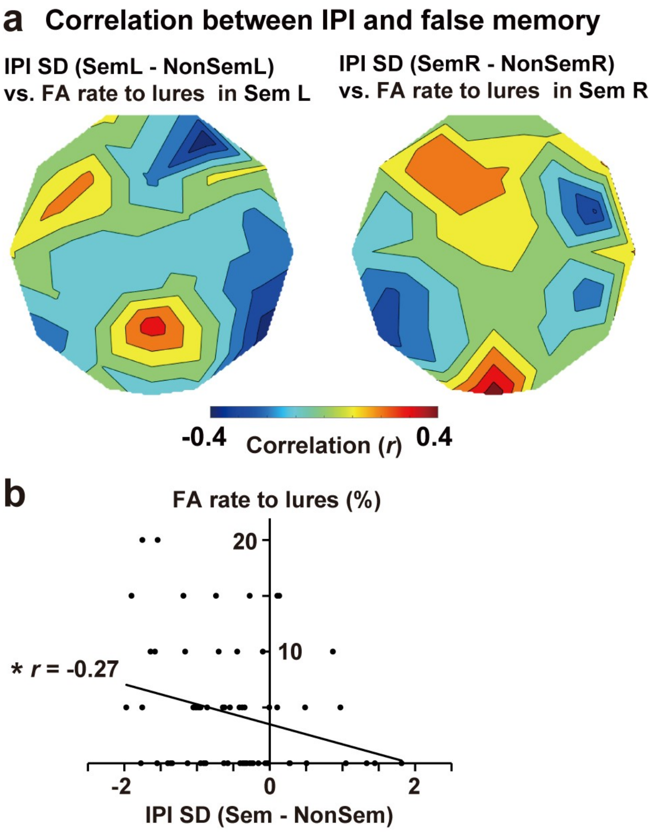

Supplementary Figure 1. Correlation between IPI and behavioral data (false memory) in Experiment 1. (**a**) Correlation *r*-maps between a difference in IPI-SD (Sem - NonSem) and a false-alarm (FA) rate to lure probes. Negative correlations over the temporal cortex indicate that participants with a greater reduction of IPI-SD (Sem < NonSem) showed a higher rate of false memory. (**b**) Individual data. Based on results of **Figure 4c**, we selected four EEG sensors showing a significant reduction of IPI-SD in Sem compared to NonSem trials; PO4, T6, T5, and CP5. The IPI-SD in the right temporal cortex (SemL – NonsemL, average of PO4 and T6) were plotted against FA rates in SemL (N = 34). The IPI-SD in the left temporal cortex (SemR – NonsemR, average of T5 and CP5) were also plotted against FA rates in SemR (N = 34). A correlation coefficient obtained from those 68 points of data was $r = -0.27$ ($p = 0.028$). $^*p < 0.05$.

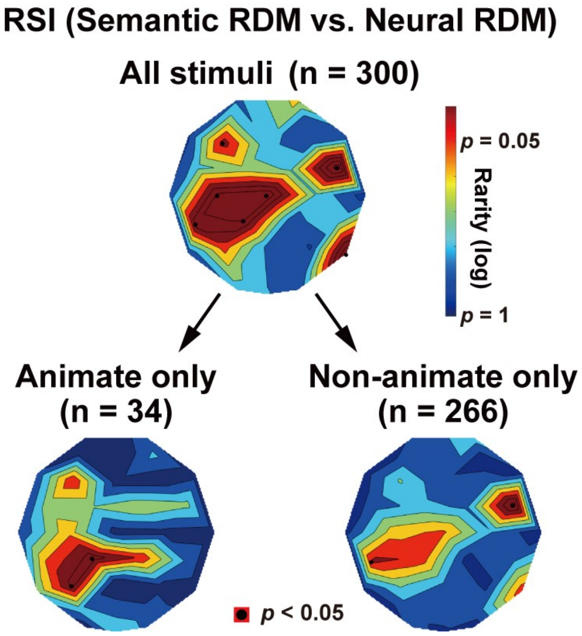

Supplementary Figure 2. Representational similarity analysis (RSA) using a subset of words in Experiment 2. Top panel: Rarity maps of RSI (representational similarity index) using the data of all 300 words (same as the left panel of **Fig. 8b**). Lower-left panel: Results of RSA using the data of 34 words representing animate objects. A smaller size of semantic and neural RDMs (34 × 34) was used in this analysis. Lower-right panel: Results of RSA using the data of 266 words representing non-animate objects. Significant RSIs were observed over the left temporal regions in all maps. The RSI in **Figure 8b (left)** thus reflect the similarity of IPIs modulated by semantic distances among words, not emerging from a task requirement (a binary distinction between animate and non-animate objects).

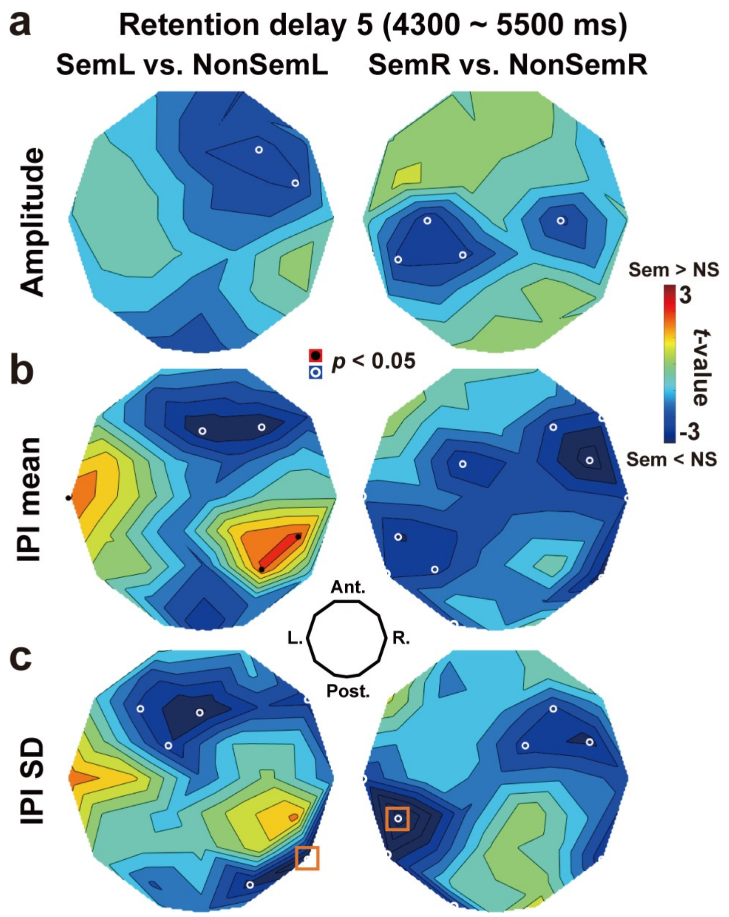

Supplementary Figure 3. Effects of semantic relatedness on the three oscillatory measures in Experiment 1. (**a**) *t*-map of oscillation amplitude. (**b**) *t*-map on mean IPIs (**c**) *t*-map on SDs of IPIs. In **Figure 4**, a pre-processing band-pass filter of 0.1 – 200 Hz was applied to the data of 28 participants, while the filter of 0.5 – 200 Hz was used for the remaining six participants. This figure shows results when the 0.5 – 200 Hz filter was applied to all 34 participants. Numbers of trials that remained after a rejection (mean ± SD, max: 60) were 53.09 ± 9.03 (SemL), 52.18 ± 9.45 (SemR), 52.21 ± 9.61 (NonSemL) and 52.24 ± 10.22 (NonSemR). A two-way ANOVA of Sem/NonSem × L/R indicated no main effect or interaction ($F(1,33) < 1.53$, $p > 0.22$, $\eta^2 < 0.044$ for all). As **Figure 4**, significant differences after the FDR correction (Sem < NonSem) were observed in IPI-SD over the posterior temporal cortex contralateral to a cued hemifield.
