# Supplementary material for: Harmonic memory signals in the human cerebral cortex induced by semantic relatedness of words
Source: NPJ Sci Learn. 2024 Feb 14;9:6. doi: 10.1038/s41539-024-00221-1 (PMC10866900; doi:10.1038/s41539-024-00221-1)
Supplement: Supplementary file 2 — Reporting summary [file 41539_2024_221_MOESM2_ESM.pdf]

nature portfolio

Corresponding author(s): Yasuki NOGUCHI

Last updated by author(s): Feb 1, 2024

Reporting Summary

Nature Portfolio wishes to improve the reproducibility of the work that we publish. This form provides structure for consistency and transparency in reporting. For further information on Nature Portfolio policies, see our Editorial Policies and the Editorial Policy Checklist.

Statistics

For all statistical analyses, confirm that the following items are present in the figure legend, table legend, main text, or Methods section.

n/a	Confirmed	
☐	☒	The exact sample size (*n*) for each experimental group/condition, given as a discrete number and unit of measurement
☐	☒	A statement on whether measurements were taken from distinct samples or whether the same sample was measured repeatedly
☐	☒	The statistical test(s) used AND whether they are one- or two-sided<br>*Only common tests should be described solely by name; describe more complex techniques in the Methods section.*
☐	☒	A description of all covariates tested
☐	☒	A description of any assumptions or corrections, such as tests of normality and adjustment for multiple comparisons
☐	☒	A full description of the statistical parameters including central tendency (e.g. means) or other basic estimates (e.g. regression coefficient) AND variation (e.g. standard deviation) or associated estimates of uncertainty (e.g. confidence intervals)
☐	☒	For null hypothesis testing, the test statistic (e.g. *F*, *t*, *r*) with confidence intervals, effect sizes, degrees of freedom and *P* value noted<br>*Give P values as exact values whenever suitable.*
☒	☐	For Bayesian analysis, information on the choice of priors and Markov chain Monte Carlo settings
☒	☐	For hierarchical and complex designs, identification of the appropriate level for tests and full reporting of outcomes
☐	☒	Estimates of effect sizes (e.g. Cohen's *d*, Pearson's *r*), indicating how they were calculated

Our web collection on statistics for biologists contains articles on many of the points above.

Software and code

Policy information about availability of computer code

Data collection	Matlab Psychophysics Toolbox. Custom codes are available by contacting the corresponding author.
Data analysis	Matlab Brainstorm Toolbox. Custom codes are available by contacting the corresponding author.

For manuscripts utilizing custom algorithms or software that are central to the research but not yet described in published literature, software must be made available to editors and reviewers. We strongly encourage code deposition in a community repository (e.g. GitHub). See the Nature Portfolio guidelines for submitting code & software for further information.

Data

Policy information about availability of data

All manuscripts must include a data availability statement. This statement should provide the following information, where applicable:

- Accession codes, unique identifiers, or web links for publicly available datasets
- A description of any restrictions on data availability
- For clinical datasets or third party data, please ensure that the statement adheres to our policy

The data that support the findings of this study are available upon request from the corresponding author (Y.N.) for non-commercial use without restriction.

Research involving human participants, their data, or biological material

Policy information about studies with human participants or human data. See also policy information about sex, gender (identity/presentation), and sexual orientation and race, ethnicity and racism.

Reporting on sex and gender	I reported the number of female participants both in Experiments 1 and 2. The information of gender is based on self reporting. Because sex or gender was not a variable of interest, I did not perform a sex- or gender-based analysis.
Reporting on race, ethnicity, or other socially relevant groupings	The information about race, ethnicity, or other socially relevant groupings was not collected because they were not a variable of interest in the present study.
Population characteristics	Thirty four healthy subjects (native speakers of Japanese) participated in Experiment 1 (age range: 18 - 42).<br>Twenty seven healthy subjects (native speakers of Japanese) participated in Experiment 2 (age range: 18 - 42).
Recruitment	Participants were students and staffs in Kobe University. They were collected via a subject-recruitment system.
Ethics oversight	The present study was approved by the ethics committee of Kobe University, Japan

Note that full information on the approval of the study protocol must also be provided in the manuscript.

Field-specific reporting

Please select the one below that is the best fit for your research. If you are not sure, read the appropriate sections before making your selection.

☒ Life sciences ☐ Behavioural & social sciences ☐ Ecological, evolutionary & environmental sciences

For a reference copy of the document with all sections, see nature.com/documents/nr-reporting-summary-flat.pdf

Life sciences study design

All studies must disclose on these points even when the disclosure is negative.

Sample size	Sample size was determined by a power analysis. The type I error rate and statistical power were set at 0.05 and 0.80, respectively. An effect size was assumed to be middle (0.5)
Data exclusions	In Experiment 1, data of one subject were excluded from analysis due to excessive noise in EEG waveforms. In Experiment 2, data of three participants were excluded from analyses because of a technical problem (loss of EEG data, N = 1) and excessive noise (N = 2).
Replication	Main results in Experiment 1 were successfully replicated in Experiment 2.
Randomization	An order of trials in experimental and control conditions was fully randomized.
Blinding	Participants did not know a purpose or hypothesis of the present study. Experimenters (technical staffs) did not know a purpose or hypothesis of the present study, either.

Reporting for specific materials, systems and methods

We require information from authors about some types of materials, experimental systems and methods used in many studies. Here, indicate whether each material, system or method listed is relevant to your study. If you are not sure if a list item applies to your research, read the appropriate section before selecting a response.

Materials & experimental systems

n/a	Involved in the study	
☒	☐	Antibodies
☒	☐	Eukaryotic cell lines
☒	☐	Palaeontology and archaeology
☒	☐	Animals and other organisms
☒	☐	Clinical data
☒	☐	Dual use research of concern
☒	☐	Plants

Methods

n/a	Involved in the study	
☒	☐	ChIP-seq
☒	☐	Flow cytometry
☒	☐	MRI-based neuroimaging

Plants

Seed stocks	*Report on the source of all seed stocks or other plant material used. If applicable, state the seed stock centre and catalogue number. If plant specimens were collected from the field, describe the collection location, date and sampling procedures.*
Novel plant genotypes	*Describe the methods by which all novel plant genotypes were produced. This includes those generated by transgenic approaches, gene editing, chemical/radiation-based mutagenesis and hybridization. For transgenic lines, describe the transformation method, the number of independent lines analyzed and the generation upon which experiments were performed. For gene-edited lines, describe the editor used, the endogenous sequence targeted for editing, the targeting guide RNA sequence (if applicable) and how the editor was applied.*
Authentication	*Describe any authentication procedures for each seed stock used or novel genotype generated. Describe any experiments used to assess the effect of a mutation and, where applicable, how potential secondary effects (e.g. second site T-DNA insertions, mosiacism, off-target gene editing) were examined.*
